# Statue of Dr. Aletta Henriëtte Jacobs (1854–1929): Physician, Activist, and an Inspiration

**DOI:** 10.1177/09677720231177293

**Published:** 2023-06-04

**Authors:** Hareesha Rishab Bharadwaj, Jack Wellington, Alexander Wellington

**Affiliations:** 1 Faculty of Biology Medicine and Health, 5292 The University of Manchester, Manchester, UK; 2 Cardiff University School of Medicine, Cardiff University, Cardiff, UK; 3 Cardiff University School of Biosciences, Cardiff University, Cardiff, UK

**Keywords:** Feminism, equal rights, women in medicine, international women's rights

## Abstract

Dr Aletta Henriette Jacobs (9 February 1854 to 10 August 1929) was a Dutch physician and advocate of modern-day women's rights, being among the first female clinicians and to formally enrol at a Dutch university. She bolstered the Dutch and international women's movements and pioneered as the first woman to develop a clinic based on contraceptive principles in 1882 internationally. Her legacy has become paramount in the progression of modern-day feminism, where her vigour for equality and diversity has stipulated campaigns to demand women's voting rights, deregulate acts of prostitution, improve working conditions for women, and promote world peace through her work.

A beautifully carved sculpture of Dr. Aletta Henriëtte Jacobs elegantly graces the entrance to the Faculty of Arts Building in Groningen. Her statue serves to immortalize her pivotal contributions towards furthering the cause of health equality and justice, in many ways, forming the foundation towards tackling the patriarchal institutions that unfortunately crippled Europe throughout the early 19th century (Figure 1).

**Figure 1.**
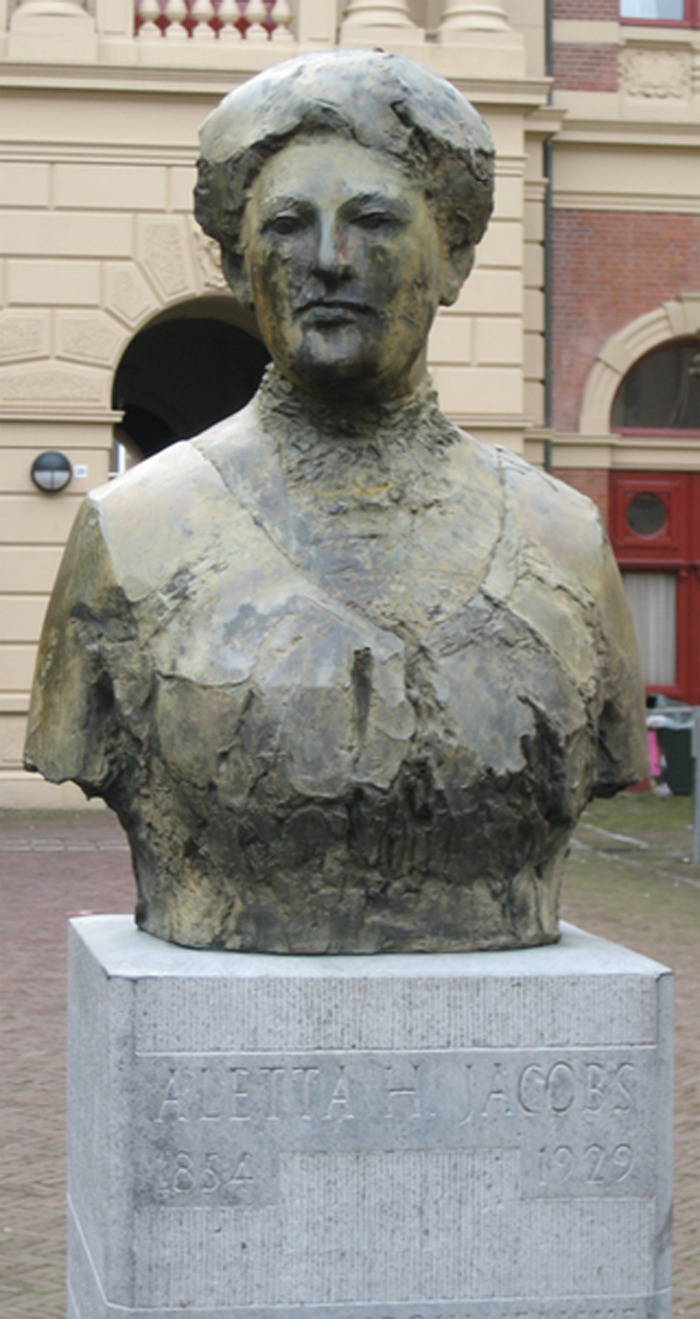
Statue of Dr. Aletta Jacobs in Groningen, Netherlands. Carved by Theresia van der Pant in 1988, it aims to serve as an everlasting testament to Dr. Aletta's contribution to furthering women's health and suffrage. Image courtesy of WikiMedia Commons.^9^

## A tumultuous beginning

Born to Abraham Jacobs and Anna de Jongh in Sappemeer, young Aletta demonstrated an early interest in medicine. Her father, who was a physician, was her idol.^1^ Unfortunately, much of the early 19th century saw very few educational opportunities for women in particular. For much of her youth, Aletta mostly stayed at home, where she indulged in learning French, German, Greek and Latin.^2^

Undoubtedly, Aletta was born during a time in which educational opportunities were severely restricted for women. Despite what seemed like a plethora of unsurmountable barriers, young Aletta's zeal for education meant that she would relentlessly rebel against societal norms to ensure that she was allowed to further her studies, just like her male peers were allowed to. Women were exclusively barred from higher education at the time.^3^ When Aletta learnt of this, she wrote to the director of Rijks Hogere Burgerschool, questioning the decision; and after relentless pursuit, she was allowed to attend high school, making her one of the only female students. After graduating, she then began to prepare for university; despite knowing that women were barred from university, she prepared for and successfully passed her entry examinations. She wrote to the Council of Ministers, requesting exclusive permission to attend university, which was provisionally granted, after a series of back-and-forth arguments. This would make Aletta the first female student at a Dutch University, the first woman to then obtain a doctorate in the Netherlands, and consequently upon graduation, the first female physician in the Netherlands.^4^

## Contributions to women's health

Dr. Jacobs’ took a note of how women at the time were severely disadvantaged, particularly when it came to accessing healthcare. Her network comprised of like-minded activists, including Elizabeth Garrett Anderson (the first female physician in England), who shared the common aim of advancing gender equality.^5^ She set up her private practice in Amsterdam; staffed exclusively by women, for women. Of particular note, she grew increasingly concerned about the lack of public-health awareness amongst women, for which she ran weekly educational sessions. She also established the first birth-control centre in the Netherlands; with the intention of combatting the growing number of unplanned pregnancies and rising rates of sexually transmitted infections. Dr. Jacobs also conducted the first contraception based clinical trial, assessing the impact of diaphragms; and would consequently strive to introduce contraception to Dutch women.^6^

## An advocate for equality

Dr. Jacobs’ efforts were not only limited to the sphere of health; in addition, she worked relentlessly to advance the status of women in society. By many, she was considered a radical. Dr. Jacobs was one of the founders of the *Vereeniging voor Vrouwenkiesrecht*, a society established to advocate for universal suffrage.^7^ In addition, she is known for literary pieces on women's economic independence, suffrage, political representation, and the legalization of prostitution, as well as translations of world-renowned feminist publications into Dutch. She also regularly attended equality and diversity themed conferences across Europe, furthering her message to the wider audience.^8^ Through this, Aletta was able to appeal to the conscience and thought of many, and spark the idea of a society based on merit and equality for all.

## Leaving behind a legacy

A true proponent of women's equality in many regards, Dr. Aletta Henriëtte Jacobs passed away on the 10^th^ of August 1929, aged 75. Her legacy remains a testament to her efforts and continues to inspire many till date.
